# Comparison of Synovial Fluid and Serum Procalcitonin for Diagnosis of Periprosthetic Joint Infection: A Pilot Study in 32 Patients

**DOI:** 10.1155/2018/8351308

**Published:** 2018-10-01

**Authors:** Paphon Sa-ngasoongsong, Siwadol Wongsak, Chavarat Jarungvittayakon, Kawee Limsamutpetch, Thanaphot Channoom, Viroj Kawinwonggowit

**Affiliations:** Department of Orthopedics, Faculty of Medicine Ramathibodi Hospital, Mahidol University, Bangkok, Thailand

## Abstract

**Background:**

Periprosthetic joint infection (PJI) remains challenging since a “gold standard” for diagnosis has not yet been established. This study aimed to evaluate the accuracy of synovial fluid procalcitonin (SF-PCT) and serum procalcitonin as a diagnostic biomarker for PJI and to compare its accuracy against standard methods.

**Methods:**

A prospective cohort study was conducted during 2015–2017 in 32 patients with painful hip or knee arthroplasty who have underwent revision surgery. Relevant clinical and laboratory data were collected. PJI was diagnosed based on the 2013 international consensus criteria. Preoperative blood sample and intraoperatively acquired joint fluid were taken for PCT measurement with a standard assay. Diagnostic accuracy was analyzed by the receiver-operating characteristic curve and the area under the curve (AUC).

**Results:**

Twenty patients (62.5%) were classified as the PJI group, and 12 (37.5%) were classified as the aseptic loosening group. The median age was 68 years (range 38–87 years). The median values of SF-PCT and serum PCT in the PJI group were both significantly higher than those in the aseptic loosening group: the median serum PCT levels (interquartile range: IQR) were 0.33 ng/mL (0.08-2.79 ng/mL) in the PJI group compared with 0.04 ng/mL (0.03-0.06 ng/mL), and the median SF-PCT levels (IQR) were 0.16 ng/mL (0.12-0.26 ng/mL) in PJI group compared with 0.00 (0.00-0.00 ng/mL) (*p* < 0.001, both). SF-PCT, with a cut-off level of 0.08 ng/mL, had an AUC of 0.87, a sensitivity of 90.0%, a specificity of 83.3%, and a negative likelihood ratio (LR-) of 0.12. Serum PCT, with a standard cut-off level of 0.5 ng/mL, had an AUC of 0.70, a sensitivity of 40.0%, a specificity of 100.0%, and a LR- of 0.60.

**Conclusion:**

SF-PCT appears to be a reliable test and could be useful as an alternative indicator or in combination with standard methods for diagnosing PJI.

## 1. Introduction

Periprosthetic joint infection (PJI) is a serious complication after total joint arthroplasty resulting in devastating consequences, such as revision surgery, limb loss, or death [1–3]. However, the diagnosis of this condition is difficult and often delayed, especially with chronic or low-grade infections, due to the lack of “gold standard” examinations and limited accuracy with current diagnostic methods. Therefore, a combination of preoperative and intraoperative markers—including synovial fluid cell count/differential, serum inflammatory markers, cultures, clinical signs, and tissue pathology—are required for PJI diagnosis. Recent studies regarding the new diagnostic techniques demonstrated that some biomarkers—such as procalcitonin (PCT), interleukin-6 (IL-6), and *α*-defensin—are helpful and a better marker for PJI [4–6]. Moreover, several studies also showed that the synovial fluid biomarkers obtained directly from the infected joint are more reliable and accurate for diagnosing PJI compared to serum biomarkers and other existing tests [7, 8].

PCT, the precursor of calcitonin, is a 116-amino-acid protein produced by the neuroendocrine and the parafollicular cells of the thyroid. Serum PCT level is generally very low (< 0.05 ng/mL) in healthy subjects [9], but specifically elevates in bacterial and fungal infections [10]. It is also unresponsive or only mildly reactive to aseptic inflammation and viral infection [11]. Therefore, numerous studies have shown its ability for differentiating septic arthritis from the aseptic condition [12–14]. Regarding the accuracy with PJI diagnosis, a recent meta-analysis showed that serum PCT had a pooled sensitivity and a pooled specificity for detecting PJI as 53% and 92%, respectively [15]. However, to the best of our knowledge, while serum PCT seems reliable [4, 15, 16], only a few studies addressed the efficacy of synovial fluid PCT (SF-PCT) for PJI diagnosis [13], and its diagnostic utility has not been clearly established. The aim of this study was to assess synovial fluid and serum levels of PCT as a diagnostic tool for PJI and to evaluate their diagnostic accuracy compared with the standard tests.

## 2. Patients and Methods

### 2.1. Study Design, Inclusion, and Exclusion Criteria

This study design was a single-centered prospective cohort study in a medical university hospital, and the study was approved by the institutional board review committee (Faculty of Medicine Ramathibodi Hospital, Mahidol University: Protocol number ID 05-58-01). All patients signed informed consent forms prior to being enrolled. The study was conducted in accordance with the declaration of Helsinki. Between 2015 and 2017, patients undergoing revision hip or knee arthroplasty were recruited into this prospective study. The patient-inclusion criteria were (1) pain at the site of total hip or total knee arthroplasty that prompted a clinical evaluation for infection or possible revision hip or knee arthroplasty, (2) no history of previous septic arthritis treatment or previous septic revision surgery, (3) sufficient synovial fluid for the study methods, and (4) sufficient clinical and laboratory data for PJI classification according to the criteria of the International Consensus Meeting on Periprosthetic Joint Infection 2013 [17] (Tables 1 and 2). Patients were excluded if they received any antibiotics or joint puncture treatments prior to enrollment in the current study.

All patients underwent standard diagnostic evaluation for PJI diagnosis. Preoperative blood samples were taken for complete blood count (CBC) erythrocyte sedimentation rate (ESR), c-reactive protein (CRP), and PCT. Joint aspiration was done intraoperatively before opening the joint capsule, and then synovial fluid was sent for cell differentiation, cell count, gram stain, aerobic culture, and PCT. Intraoperative frozen section was performed. Periprosthetic tissue from five different areas (joint capsule, synovial lining, intramedullary material, granulation tissue, and bone fragments) was delivered for microbiology and histology.

### 2.2. Determination of the Levels of Serum and Synovial Fluid PCT

PCT levels were quantified using a standard quantitative PCT enzyme immunoassay kit, according to the manufacturers' instructions (Elecsys® BRAHMS PCT test, Roche Diagnostics Ltd., Switzerland), on the Roche Cobas e601 analyzer. The lower limit of detection was 0.02 ng/mL. The specimens, either blood or synovial fluid, were collected and kept at room temperature (10°C–25°C) and were measured within 2 hours. When synovial fluid cannot be measured within 2 hours, the specimen must be kept at approximately 2°C–8°C and must be measured within 24 hours. Due to the high viscosity of synovial fluid, the specimen was diluted at a ratio of 1:4 (100 *µ*L of synovial fluid sample with 300 *µ*L normal saline). Therefore, the synovial PCT level was then calculated from the measured PCT value multiplied by 4, such as 0.08, 0.12, and 0.16 ng/mL.

### 2.3. Statistical Analysis

Statistical analysis was carried out with MedCalc Statistical Software version 15.8 (MedCalc Software bvbv, Ostend, Belgium). Normally distributed continuous data were shown as mean ± standard deviation (SD) and compared using student's* t*-test. Non-normally distributed continuous data were shown as median (interquartile range [IQR]) and compared using the Mann–Whitney U test. The categorical variables were presented as number of cases with proportion and compared with Chi-square or Fisher's exact test. A *p* value < 0.05 was considered statistically significant. Diagnostic accuracy of serum PCT and SF-PCT was assessed with receiver-operating characteristic (ROC) curves between the PJI and aseptic loosening groups. Sensitivity, specificity, positive likelihood ratio (LR+), negative likelihood ratio (LR-), area under the ROC curve (AUC), and their 95% confidence interval (CI) for any cut-off level were calculated.

## 3. Results

A total of 32 patients (5 revision hip arthroplasties and 27 revision knee arthroplasties) were recruited into our prospective study between 2015 and 2017. Regarding the International Consensus Criteria on PJI [17], 20 patients (20 revision knee arthroplasties) were classified in the PJI group and 12 patients (5 revision hip arthroplasties and 7 revision knee arthroplasties) were classified in the aseptic group. The patient characteristics data are presented in Table 3. There were 7 males (22%) and 25 females (78%). The median patient age was 68 years (range 38–87 years). The mean BMI was 26.9 ± 4.0 kg/m^2^, and the median CCI was 3 (range 0–9). Of these, 2 patients had preexisting rheumatoid arthritis (1 patient in each group) and were receiving immunomodulating drugs. No significant difference existed in age, BMI, operated side, CCI, presence of systemic inflammatory disease, and concomitant immunomodulation drugs between both groups. However, the PJI group was significantly higher for male gender, revision knee arthroplasties, body temperature, and serum WBC count than the aseptic group (*p* < 0.05 all).

Tables 4 and 5 demonstrate the relevant clinical and laboratory findings according to the PJI definition [17] in both groups and the microbiological findings in our study. Of the 20 patients with PJI, 13 (65%) had positive synovial fluid culture and 14 (70%) had positive tissue culture. The most common microorganism from cultures-positive PJI was* Streptococci *(n = 7, 50%). The PJI group demonstrated significantly greater values in serum and synovial fluid markers related to infection than the aseptic loosening group (*p* < 0.001, all). The median serum PCT level (interquartile range: IQR) in the PJI and aseptic groups was 0.33 (0.08 to 2.79) and 0.04 (0.03 to 0.06), respectively (*p* < 0.001). The median SF-PCT (IQR) in the PJI and aseptic groups was 0.16 (0.12 to 0.26) and 0.00 (0.00 to 0.00), respectively (*p* < 0.001). Regarding the aseptic loosening group, the median serum PCT and SF-PCT values from revision hip arthroplasties (0.04 and 0.00 ng/mL) did not significantly differ compared to those from revision knee arthroplasties (0.04 and 0.00 ng/mL) (*p* = 0.400 and 0.287, respectively).

**Figure 1:**
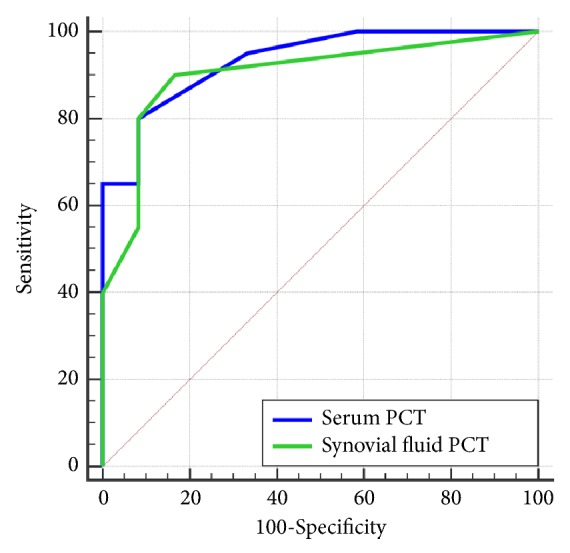
Receiver-operating characteristic (ROC) curves comparing ability between serum and synovial fluid PCT to detect periprosthetic joint infection (PJI) before revision arthroplasty.

Table 6 and Figure 1 show the diagnostic accuracy of PJI diagnosis by using serum PCT or SF-PCT in each cut-off value and ROC curve comparison between serum PCT and SF-PCT. The cut-off references of serum PCT were set as 0.1, 0.3, and 0.5 ng/mL, whereas those of SF-PCT were set as 0.08, 0.12, and 0.16 ng/mL. Regarding the accuracy of the serum PCT test with the standard cut-off reference level as 0.5 ng/mL, the sensitivity, specificity, LR+, and LR- were 40.0%, 100.0%, not available, and 0.60, respectively. However, with the lower cut-off level as 0.1 ng/mL, the serum PCT test showed sensitivity, specificity, LR+, and LR- as 65.0%, 91.7%, 7.80, and 0.38, respectively. The AUC of 0.5 and 0.1 ng/mL cut-off levels was 0.70 and 0.78, respectively.

Regarding the accuracy of the SF-PCT test for PJI diagnosis, the cut-off value as 0.08 ng/mL resulted in sensitivity of 90.0%, specificity of 83.3%, LR+ of 5.40, and LR- of 0.12. Conversely, the higher cut-off level as 0.12 ng/mL showed sensitivity of 80.0%, specificity of 91.7%, LR+ of 9.40, and LR- of 0.22. The AUC of 0.08 and 0.12 ng/mL cut-off levels was 0.87 and 0.86, respectively (Table 6).

## 4. Discussion

Periprosthetic joint infection (PJI) is one of the most severe and costly complications following total joint arthroplasty. Although there is an international consensus for the definition of PJI, no single “gold standard” test currently exists for diagnosing PJI. Recently, many studies have reported the usefulness of synovial fluid cytokines—such as interleukin-6, c-reactive protein, and alpha-defensin—as alternative and better diagnostic markers for PJI compared to the standard technique [7, 8, 18–21]. The overall sensitivity and specificity of these markers were more than 80% and 90%, respectively [7]. However, to our knowledge, although some biomarkers have demonstrated excellent diagnostic performance for PJI, the comparison of diagnostic accuracy between these biomarkers did not achieve statistical significance [8]. Moreover, according to the current evidence on these new biomarkers, serum PCT is a promising and reliable test, but the utility of synovial fluid PCT for detecting PJI has not been clearly demonstrated.

The results of this study show that both serum PCT and SF-PCT could be used as diagnostic biomarkers to support clinicians in differentiating PJI from aseptic loosening. The PJI group had significantly higher serum PCT and SF-PCT values, the same as serum ESR and CRP (*p* < 0.001 all), compared with the aseptic loosening group (Table 4). Using ROC curve analysis, the present study demonstrates that serum PCT, with the standard cut-off level as 0.5 ng/mL (a sensitivity of 40%, a specificity of 100%, and AUC of 0.70), is comparable to PCT from the previous meta-analysis (pooled sensitivity of 53%, pooled specificity of 92%, and AUC of 0.76) [15]. Additionally, this study also reveals that, with the lower serum PCT cut-off level as 0.1 ng/mL, the diagnostic accuracy of serum PCT could be further improved to sensitivity of 65%, persistently good specificity (92%), and AUC of 0.78 (Table 5). However, serum PCT with a lower cut-off level should be used with caution and may need future larger studies to ensure an effective strategy implementation.

Regarding ROC curve analysis, SF-PCT showed an ability to be a more valuable biomarker for identifying PJI from aseptic loosening than serum PCT. With a cut-off level as 0.08 ng/mL, SF-PCT showed the greatest accuracy with sensitivity of 90%, specificity of 83%, LR+ of 5.40, LR- of 0.12, and AUC of 0.87 (Table 5). These high sensitivity, high LR+, and very low LR- characteristics are all good indicators for ruling in and out the diagnosis of PJI [22], especially for preoperative and intraoperative settings. This is because the PCT test would take only 30 minutes to perform in the laboratory and may have the potential to become a point-of-care test in the patients who obtain the synovial fluid. Additionally, this study also found that, according to the PJI group, the concentration of PCT in blood (median value 0.33 ng/mL, interquartile range 0.08 ng/mL–2.79 ng/mL) seemed to be greater (about two times) than those in joint fluid (median value 0.16 ng/mL, interquartile range 0.12 ng/mL–0.26 ng/mL). However, the difference did not reach statistical significance (*p* = 0.20) (Table 4). This could imply that the cut-off reference for SF-PCT should be different from those for serum PCT, the same as the other synovial fluid biomarker [21].

Concerning the comparison of the diagnostic performance between synovial fluid biomarkers for PJI diagnosis, although this study demonstrated a good accuracy of SF-PCT for PJI diagnosis with an AUC of 0.87, this diagnostic accuracy appeared to be slightly inferior to biomarkers from previous studies—such as CRP, IL-6, and alpha-defensin—with AUC between 0.90 and 0.99 [18, 20, 23, 24]. However, due to the previously noted potential of the PCT test, we still recommend using SF-PCT as a complementary tool with the standard technique for diagnosing PJI.

This study also had some limitations. Firstly, due to the prospective cohort nature in only one university hospital center, our sample size was relatively small and included both knee and hip patients. Therefore, future longitudinal studies with a larger sample size and a specific analytic approach for revision knee or hip arthroplasties would require determining the usefulness of SF-PCT for detecting PJI. Secondly, this study did not include patients with prior antibiotics therapy or with concomitant disease that might affect SF-PCT, such as crystal-induced arthritis or malignancy [13]. Lastly, a diagnostic accuracy comparison between SF-PCT and other biomarkers was not performed. However, the information related to other biomarkers is already published.

## 5. Conclusion

The accuracy of SF-PCT was significantly higher than that of serum PCT. Therefore, SF-PCT may be used as an alternative indicator in the differential diagnosis of PJI from aseptic loosening in cases where patients are undergoing revision hip or knee arthroplasty. However, further prospective studies with a larger sample size are required to validate the usefulness of SF-PCT.

## Figures and Tables

**Table 1:** Definition for periprosthetic joint infection by International Consensus Group.

Periprosthetic joint infection (PJI) is present when one major criterion exists or when three of five minor criteria exist	
Major criteria	(1) Two positive periprosthetic cultures with phenotypically identical organisms
	(2) A sinus tract communicating with the joint
Minor criteria	(1) Elevated serum C-reactive protein AND erythrocyte sedimentation rate
	(2) Elevated synovial fluid white blood cell (WBC) count OR ++change on leukocyte esterase test strip
	(3) Elevated synovial fluid polymorphonuclear neutrophil percentage
	(4) Positive histological analysis of periprosthetic tissue
	(5) A single positive culture

**Table 2:** The threshold for the minor diagnostic criteria according to the International Consensus Group.

**Criterion **	**Acute PJI (<90 days) **	**Chronic PJI (>90 days)**
**Erythrocyte sedimentation rate (mm/hr) **	Not helpful; no threshold was determined	30
**C-reactive protein (mg/L) **	100	10
**Synovia white blood cell count (cells/** **µ** **L)**	10,000	3,000
**Synovial polymorphonuclear percentage (**%**) **	90	80
**Leukocyte esterase **	+ or ++	Same as acute
**Histological analysis of tissue **	>5 neutrophils/HPF in 5 HPFs (x400)	Same as acute

PJI: periprosthetic joint infection; HPF: high power field.

**Table 3:** Patient characteristics data.

	**Total**	**PJI**	**Aseptic loosening**	**p value**
	**(n=32)**	**(n=20)**	**(n=12)**	
Age, year◆	68 (65–74)	70 (66–76)	67 ± 5	0.098
Female gender *◎*	25 (78%)	13 (65%)	12 (100%)	0.029^*∗*^
BMI, kg/m^2^△	26.9 ± 4.0	26.3 ± 4.5	28.1 ± 3.0	0.229
Hip : Knee❖	5 : 27	0 : 20	5 : 7	0.004^*∗*^
Left : Right❖	14 : 18	10 : 10	4 : 8	0.471
CCI◆	3 (2-4)	3 (3-4)	2.5 ± 1.0	0.083
Systemic inflammatory disease*◎*	2 (6%)	1 (5%)	1 (8%)	1.000
Receiving immunomodulating drugs*◎*	2 (6%)	1 (5%)	1 (8%)	1.000
Body temperature (°C)◆	36.8 (36.5–37.3)	37.4 ± 0.9	36.6 ± 0.2	0.010^*∗*^
WBC count (cell/mm^3^)◆	6,620 (5,755–10,250)	9,170 (1,700–28,100)	6106 ± 1433	0.021^*∗*^

PJI: periprosthetic joint infection, BMI: body mass index, CCI: Charlson comorbidity index, and WBC: white blood cell; ◆: value presented as mean ± SD or mean (IQR) and calculated with Mann–Whitney U test; *◎*: value presented as number of cases (percentage) and calculated with Fisher exact test or Chi-square test as appropriate; △: value presented with mean ± standard deviation and calculated with unpaired t-test; ❖: value presented as the proportion of cases with that condition and calculated with Fisher exact test; ^*∗*^significant p value with p < 0.05.

**Table 4:** Relevant clinical and laboratory findings for PJI definition.

	**PJI (n=20)**	**Aseptic loosening (n=12)**	**p value**
**Sinus tract presence*◎***	0 (0%)	0 (0%)	1.00
**Synovial fluid culture*◎***	13 (65%)	0 (0%)	<0.001
**Tissue culture*◎***	14 (70%)	0 (0%)	<0.001
**Serum markers**			
** ESR, mm/hour △▽**	81 ± 27	18.5 (12.5–36.5)	<0.001
** CRP, mg/dL △**	149 ± 98	2.9 ± 2.5	<0.001
** Serum PCT, ng/mL ▽**	0.33 (0.08–2.79)	0.04 (0.03–0.06)	<0.001
** Hip**	n/a	0.04 (0.02–0.06)	n/a
** Knee**	0.33 (0.08–2.79)	0.04 ± 0.04	0.001
**Synovial fluid markers**			
** Synovial fluid WBC, cell/mm** ^**3**^ **▽△**	78,920 (3,420–335,400)	1350 ± 827	<0.001
** **%**Neutrophil △**	90.8 ± 6.2	54.9 ± 17.2	<0.001
** SF-PCT, ng/mL▽**	0.16 (0.12–0.26)	0.00 (0.00–0.00)	<0.001
** Hip**	n/a	0.00 0.00	n/a
** Knee**	0.16 (0.12–0.26)	0.00 (0.00–0.06)	0.004

*◎*: value presented as number of cases (percentage), △: value presented as mean ± standard deviation, ▽: value presented as median (interquartile range), and n/a: not available.

**Table 5:** Microbiological findings of culture-diagnosed PJI among 14 episodes.

**Micro-organisms**	**No. (**%**)**
Gram positive	
*Staphylococcus aureus*	1 (7%)
CNS	1 (7%)
Streptococci	7 (50)
Gram positive	
*Escherichia coli*	3 (21%)
*Proteus mirabilis*	1 (7%)
Other^a^	1 (7%)

CNS: *coagulative negative staphylococcus*; ^a^: *Propionibacterium *spp.

**Table 6:** Diagnostic accuracy of PJI diagnosis using serum or synovial fluid procalcitonin.

	**Sensitivity**	**Specificity**	**AUC**	**LR+**	**LR-**
**Serum PCT (ng/mL) **					
0.1	65.0 (40.8–84.6)	91.7 (61.5–99.8)	0.78 (0.60–0.91)	7.80 (1.16–52.35)	0.38 (0.21–0.71)
0.3	50.0 (27.2–72.8)	100.0 (73.5–100.0)	0.75 (0.57–0.89)	n/a	0.47 (0.29–0.76)
0.5	40.0 (19.1–64.0)	100.0 (73.5–100.0)	0.70 (0.51–0.85)	n/a	0.60 (0.42–0.86)
**Synovial fluid PCT (ng/mL)**					
0.08	90.0 (68.3–98.8)	83.3 (51.6–97.9)	0.87 (0.70–0.96)	5.40 (1.51–19.30)	0.12 (0.03–0.46)
0.12	80.0 (56.3–94.3)	91.7 (61.5–99.8)	0.86 (0.69–0.96)	9.60 (1.45–63.50)	0.22 (0.09–0.53)
0.16	55.0 (31.5–76.9)	91.7 (61.5–99.8)	0.73 (0.55–0.87)	6.60 (0.97–44.93)	0.49 (0.29–0.82)

AUC: area under curve, LR+: positive likelihood ratio, LR-: negative likelihood ratio, and PCT: procalcitonin.

## Data Availability

The data used to support the findings of this study are available from the corresponding author upon request.
